# Goodpasture's Syndrome and p-ANCA Associated Vasculitis in a Patient of Silicosiderosis: An Unusual Association

**DOI:** 10.1155/2014/398238

**Published:** 2014-10-02

**Authors:** Amanjit Bal, Ashim Das, Dheeraj Gupta, Mandeep Garg

**Affiliations:** ^1^Department of Histopathology, Post Graduate Institute of Medical Education & Research (PGIMER), Sector 12, Chandigarh 160012, India; ^2^Department of Pulmonary Medicine, Post Graduate Institute of Medical Education & Research (PGIMER), Sector 12, Chandigarh 160012, India; ^3^Department of Radiodiagnosis, Post Graduate Institute of Medical Education & Research (PGIMER), Sector 12, Chandigarh 160012, India

## Abstract

*Introduction.* Goodpasture's syndrome is a rare clinical entity and is characterized by circulating autoantibodies which are principally directed against the glomerular/alveolar basement membrane. The etiology of Goodpasture's syndrome is still unknown. Lung involvement occurs as a result of lung injury and the exposure of new epitopes to the immune system. Recently, several studies have suggested the role of silica as one of etiological factors in ANCA associated vasculitis and glomerulonephritis. *Materials and Methods.* We present a case of a 40-year-old welder with silicosiderosis, who developed anti-GBM disease with p-ANCA positivity. *Case Report.* Patient presented to an emergency with gradually increasing breathlessness along with renal failure and died after short hospital stay. Autopsy pathology findings revealed crescentic glomerulonephritis with linear glomerular basement membrane antibody deposition, splenic vasculitis, pulmonary haemorrhage, and pulmonary silicosiderosis. *Conclusion.* This case reinforces the role of environmental triggers like exposure to silica, metal dust, and tobacco in pathogenesis of Goodpasture's syndrome and p-ANCA associated vasculitis.

## 1. Introduction

Antiglomerular basement membrane antibody disease is a rare cause of pulmonary renal syndrome and is defined by the presence of serum anti-GBM antibody. The clinical presentation is of acute rapidly progressive glomerulonephritis (RPGN) with biopsy findings of severe crescentic glomerulonephritis (GN) and a linear deposition of IgG along the GBM as evidenced by immunofluorescence (IF) [1]. When accompanied by pulmonary involvement, it is referred to as anti-GBM disease or “Goodpasture syndrome.” A positive ANCA serology, especially anti-MPO, has been identified in approximately a third of the patients with anti-GBM disease. The prognosis of dual-positive patients is comparable to patients with isolated anti-GBM nephritis. However, similar to isolated ANCA associated disease, these dual-positive cases have higher frequency of active relapses [2, 3].

The aetiology of anti-GBM disease is not known; however like other autoimmune diseases environmental triggers like exposure to hydrocarbons and crystalline silica have been implicated in its pathogenesis. Silicosis and mineral dust pneumoconiosis have been linked to an increase in autoantibodies, immune complexes, and excess production of immunoglobulins, even in the absence of a specific autoimmune disease [4].

We report a case of a 40-year-old welder with silicosiderosis, who developed anti-GBM disease with p-ANCA positivity.

## 2. Case Presentation

### 2.1. Case History

A 40-year-old male presented to emergency with gradually increasing shortness of breath of 1-month duration. One month back, patient had history of swelling all over the body, which was initially over the face and became generalized subsequently. He had cough with mucoid expectoration for the past 15 days along with streaky hemoptysis. He had decreased urine output and dark coloured urine since 5 days. He also had low grade fever for 15 days and a history of vesicular eruptions over the right mammary area since 15 days for which skin consultation was taken and was diagnosed as herpes zoster. He had a history of atypical chest pain (angina) being managed with antiplatelet and statins for past 2 years. He was a welder by occupation and used to smoke Bidi (Indian cigarette with variable amounts of tobacco), one packet per day for 12–15 years.

### 2.2. Clinical Examination and Investigations

On examination he had pallor and pedal edema. Chest auscultation revealed bilateral coarse crepitations and bronchial breathing in left axillary region. Cardiovascular system and central nervous system examination was within normal limits. ECG showed ST ↓ inferior/lateral leads and a poor progression of “R” v1–v3. Urine routine examination revealed 4+ albumin, 12–15 red blood cells, and 2–4 Pus cells. The hemolytic workup was negative. Serum CPKMB was 11 U/L and LDH was 754.8 U/L. Immunofluorescence (IF) on ethanol-fixed neutrophils showed perinuclear pattern of ANCA (pANCA, +++). Enzyme-linked immunosorbent assay (ELISA) was positive for myeloperoxidase antibodies (pANCA, Euroimmun Kit) but negative for antiproteinase 3 antibodies (cANCA) and antiglomerular basement membrane (anti-GBM) antibodies. Hepatitis B and C serologies were negative. Laboratory investigations are detailed in Table 1.

### 2.3. Radiology Findings

On ultrasound abdomen, there was mild ascites, liver was 13.3 cm, and spleen was 10.5 cm in span. Both kidneys showed increased echotexture. Chest X-ray showed bilateral diffuse alveolar shadows. CT scan showed bilateral diffuse areas of consolidation, minimal pleural effusion, and pericardial effusion.

### 2.4. Course and Management

On admission a possibility of pulmonary renal syndrome; ANCA associated vasculitis was kept. He received hemodialysis and two days later had a cardiorespiratory arrest from which he was revived and given ventilatory support. Endotracheal (ET) secretions were hemorrhagic; thus with a possibility of diffuse alveolar haemorrhage, he was started on intravenous methyl prednisone pulse and received a plasmapheresis. With pANCA positive he received injection cyclophosphamide. He was taken up for second plasmapheresis and had massive ET bleeding and cardiac arrest during plasmapheresis from which he was revived and shifted to ventilatory support. However he continued to have high oxygen requirement and had another cardiac arrest from which he could not be revived. A complete autopsy was performed after obtaining written consent from the relatives.

## 3. Autopsy Findings

**Figure 1:**
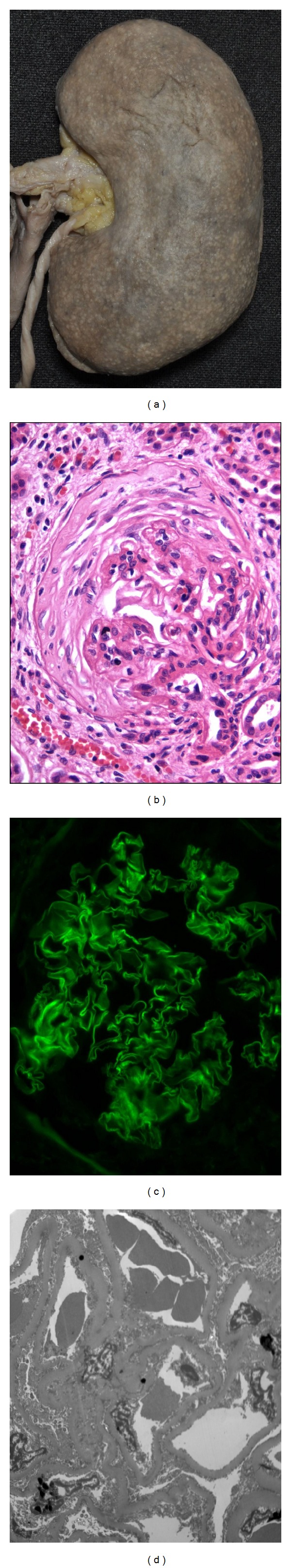
Photomicrograph showing (a) swollen and flea bitten appearance of kidney, (b) glomeruli showing crescent formation (H&E, ×400), (c) linear staining of glomerular basement membrane for IgG on immunofluorescence, and (d) no electron dense deposits seen on ultrastructural examination.

On opening the pleural cavities showed dense adhesions, the pericardial cavity yielded 200 mL of serous fluid and peritoneal cavity yielded 1.5 L of straw colored fluid. Grossly both the kidneys were swollen and had tiny red dots scattered over cortical surface giving flea bitten appearance (Figure 1(a)). On light microscopic examination, about 90% glomeruli showed crescent formation of similar age (Figure 1(b)). Majority of the crescents were fibrous or fibrocellular. Underlying glomerular tuft showed segmental sclerosis with adhesions to the Bowman's capsule. A few preserved glomeruli showed segmental fibrinoid necrosis characterized by deeply acidophilic material replacing normal architecture. Intact tufts had capillary walls of normal thickness and no evidence of endocapillary proliferation. On immunofluorescence (IF), linear staining of glomerular basement membrane for IgG (Figure 1(c)) along with C3 (3+) was seen and was negative for IgA and IgM. IF intensity was scored semiquantitatively (on a scale of 0 absent, 1+ weak, 2+ moderate, and 3+ strong). On ultrastructural examination no electron dense deposits were seen (Figure 1(d)). The tubules showed hyaline and RBC casts along with focal areas of tubular atrophy and interstitial fibrosis.

**Figure 2:**
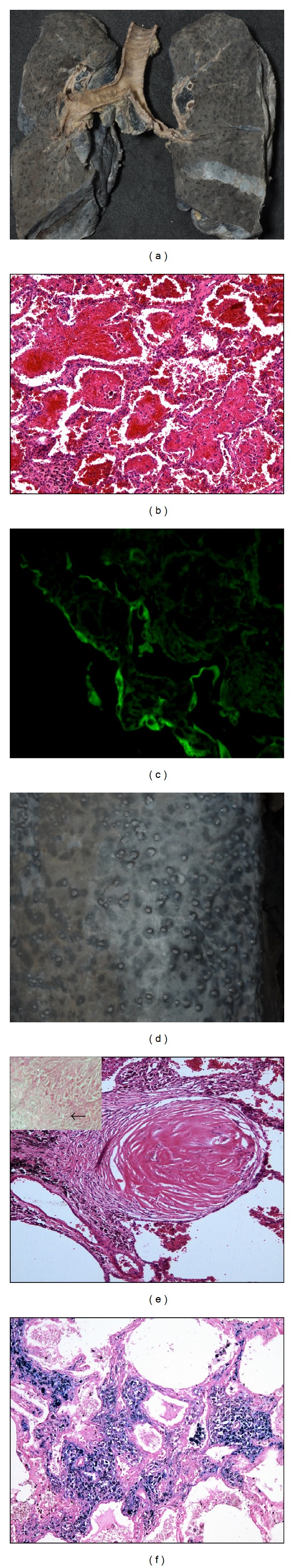
Photomicrograph showing (a) voluminous, heavy, and subcrepitant lungs which on cut surface showed haemorrhagic consolidation, (b) dominantly fresh alveolar hemorrhages (H&E, ×200), (c) linear IgG deposits on alveolar lining and capillary basement membrane by immunofluorescence, (d) pleura with numerous 0.2-0.3 cm white nodules scattered throughout the surface, (e) interstitial nodules well demarcated from adjacent lung parenchyma characterized by concentrically arranged fibroblasts and hyalinized lamellated arrangement of the collagen bundles (inset showing small polarizable refractile oval to needle shaped particles within the nodules) (H&E, ×100), (f) Perl's stain highlighting the iron content in dust macules in peribronchiolar and perivascular location composed of macrophages containing brown black particles (Perl's stain, ×200).

Both the lungs were voluminous, heavy, and subcrepitant. Pleura were dull with fibrinous tags. Cut surface showed haemorrhagic consolidation involving all lobes of both the lungs (Figure 2(a)). Hilar nodes were enlarged. Light microscopy dominantly showed fresh alveolar haemorrhages (Figure 2(b)). Interalveolar interstitium was widened by neutrophilic infiltrate which was masked by extensive haemorrhage; however no foci of leukocytoclasia were seen. Perl's stain highlighted a few intra-alveolar hemosiderin laden macrophages. On immunofluorescence alveolar lining and capillary basement membrane showed 2+ linear IgG deposits (Figure 2(c)). On ultrastructural examination no electron dense deposits were seen. Grossly the pleural surface showed numerous 0.2-0.3 cm white nodules scattered throughout the surface of both the lungs (Figure 2(d)). Microscopically these subpleural and interstitial nodules well demarcated from adjacent lung parenchyma characterized by concentrically arranged fibroblasts and hyalinized lamellated arrangement of the collagen bundles were seen (Figure 2(e)). Small polarizable refractile oval to needle shaped particles were present within the nodules (Figure 2(e), inset). In addition there were dust macules in peribronchiolar and perivascular location composed of macrophages containing brown black particles. Perl's stain highlighted the iron content in macules (Figure 2(f)). Hilar lymph nodes showed similar lamellated fibrotic nodules.

**Figure 3:**
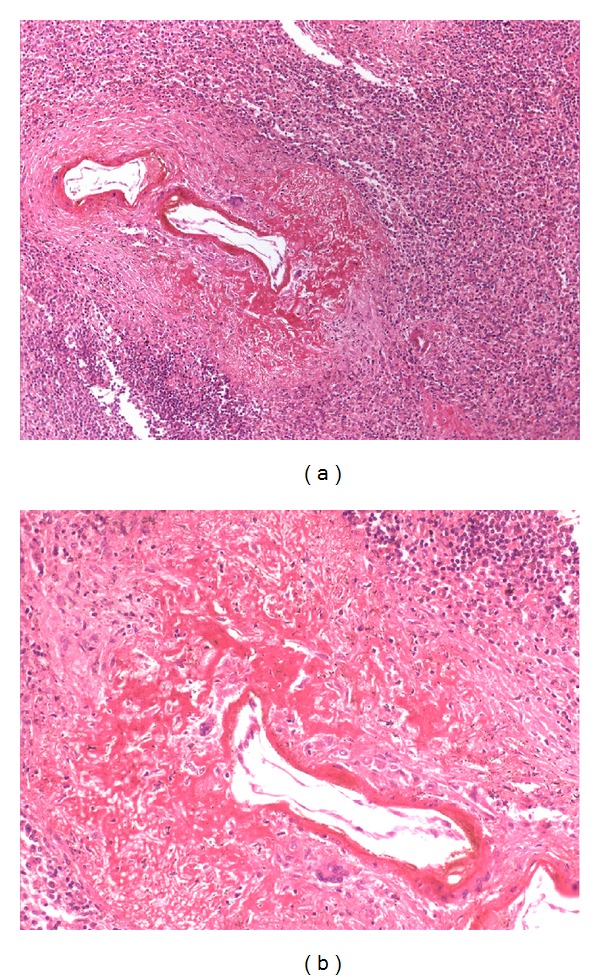
Photomicrograph showing fibrinoid necrosis of the wall of trabecular vessels of the spleen along with occasional giant cells indicative of vasculitis (H&E, ×200, ×400).

Spleen had thickened capsule and was diffluent with perisplenitis. The cut surface was congested and no focal lesions were noted. On microscopic examination, the trabecular vessels show fibrinoid necrosis of their walls along with occasional giant cells indicative of vasculitis (Figures 3(a) and 3(b)). Liver showed preserved lobular architecture; however there were randomly distributed foci of haemorrhagic hepatocytic necrosis. Periphery of these necrotic foci shows multinucleate giant cells and cells with basophilic nuclear inclusions consistent with Herpes Zoster inclusions. The heart was grossly enlarged and globular with left sided prominence and revealed old healed infarct in posterior wall and interventricular septum. Coronaries showed atherosclerotic changes with >75% occlusion in the left coronary artery (LCA), the right coronary artery (RCA) at the terminal end, and the left circumflex artery (LCX).

Based on these findings the final autopsy diagnosis of Goodpasture's syndrome and pANCA associated small vessel vasculitis in spleen with extensive silicosiderosis lung was made. In addition there were Herpes Zoster hepatitis and old healed myocardial infarction of RCA territory with triple vessel coronary atherosclerosis.

## 4. Discussion

Goodpasture's syndrome is a rare clinical entity with a prevalence of less than 1 case per million population [5]. However, pathological studies presented that anti-GBM disease is responsible for 1 to 5% of all types of antibody-induced glomerulonephritis and is the cause for 10 to 20% of crescentic glomerulonephritis cases [6]. The hallmark of anti-GBM disease is circulating autoantibodies which are principally directed against the glomerular/alveolar basement membrane. The antibodies are targeted against the type IV collagen, and the specific epitope is the NC1 domain of the alpha 3 chain. However, binding antibodies against the alpha 5 (IV) and alpha 4 (IV) chain have also been detected [7]. Pathologically crescentic glomerulonephritis and linear IgG deposits along the glomerular capillaries and/or the distal tubules detected by immunofluorescence (IF) are pathognomonic for anti-GBM disease. Pulmonary lesions present histologically as hemorrhages, with numerous hemosiderin-containing macrophages, and prominence of type II pneumocytes. Necrosis of alveolar walls with polymorphonuclear cell infiltration can also be detected; however no capillaritis is seen. On IF examination linear binding of IgG is usually detected along the alveolar basement membrane. The present case with a negative serologic assay and an autopsy specimen showing anti-GBM disease can be explained by limitation of the serologic tests. Anti-GBM antibodies were looked for by ELISA which can be spuriously negative or the levels of antibodies in circulation were very low which were below the sensitivity of the ELISA. Salama et al. [8] reported that it is possible to prove the existence of low titer anti-GBM antibody using the Biosensor analysis (biomolecular interaction analysis system) for patients with anti-GBM disease. When serum assays are negative, as in this case, renal biopsy is recommended for all patients with renal involvement because light microscopy and immunofluorescence studies can assess the extent and activity of glomerulonephritis [9].

In the present case, patient had p-ANCA positive; however there were no clinical signs of systemic vasculitis. The only evidence of p-ANCA associated vasculitis was seen on autopsy in the splenic blood vessels. Approximately 10–38% of patients with anti-GBM antibody disease are also ANCA positive (antimyeloperoxidase, or p-ANCA) and may have signs of systemic vasculitis [10]. Patients with dual antibodies are considered to be a vasculitis-variant of anti-GBM antibody nephritis. Such patients may not have a typical presentation of pulmonary-renal syndrome, resulting in delay of the correct diagnosis and initiation of treatment [11]. In a rat model it has been demonstrated that autoantibodies to MPO severely aggravate subclinical anti-GBM disease and suggest that ANCAs may be a pathogenic factor in rendering mild glomerular injury into clinically overt disease [12]. The detection of ANCA is clinically relevant, as these patients have treatable disease than those who have only anti-GBM antibodies. However, larger series suggest that the initial outcome is similar to those with anti-GBM disease alone.

The etiology of Goodpasture's syndrome is still unknown. Lung involvement occurs in patients who smoke, such as this patient, presumably because of lung injury and the exposure of new epitopes to the immune system. Development of the disease following crystalline silica exposure has also been reported in the case reports [13–16]. Other exposures have also been implicated as causal factors, including hydrocarbons, cocaine use, hard metal dust, and* D*-penicillamine [17–20]. The circumstances of our case, a welder with a history of tobacco use, suggest inhalation of metal dust and silica as the salient factors.

Recently, several studies have been published suggesting the role of silica as one of etiological factors in ANCA associated vasculitis and glomerulonephritis [21–23]. Silicosis and mineral dust pneumoconiosis have been linked to an increase in autoantibodies, immune complexes, and excess production of immunoglobulins, even in the absence of a specific autoimmune disease. Formation of ANCA as the result of silica exposure may be explained by the fact that compounds containing silica are potent stimulators of immune reaction. Silicon may induce apoptosis of monocytes and macrophages. These cells then release proteolytic enzymes, which directly damage the tissue ANCA and other autoantibodies are formed [21]. The majority of patients of silicosis had p-ANCA which is much more promiscuity marker than c-ANCA and is associated with a variety of autoimmune and inflammatory conditions.

Crystalline silica has been recognized as both a pneumotoxin and nephrotoxin. Crystalline silica particles are ingested by alveolar macrophages and result in inflammation and activation of fibroblasts [24]. This process is repeated and leads to chronic immune activity and fibrosis. Studies have shown that crystalline silica can be mobilized from the lungs to other organs, including lymph nodes, spleen, and kidney. In silicotic patients, a fourfold increase of silicon concentration was detected in the kidney tissue. Several authors described glomerulonephritis and/or kidney failure in some patients with silicosis [21]. This patient, who was a smoker, worked as an arc welder without any proper safety equipment including respirators or gloves. The details of other environmental exposures during his career as a welder are unknown.

This case reinforces the role of environmental triggers like exposure to silica, metal dust, and tobacco in pathogenesis of Goodpasture's syndrome and p-ANCA associated vasculitis. This case illustrates the complex interrelations of exposure to substances with antigenic properties and immune defects.

## Figures and Tables

**Table 1:** Laboratory investigations.

Haemoglobin	4.8 g/dL

Total leucocyte count	6400/mm^3^

Differential leucocyte count (N/L/M/E)	74/20/5/1

Platelets	81,000/mm^3^

Urine routine examination	4+ albumin,
	12–15 red blood cells
	2–4 Pus cells.

Renal function tests	Blood Urea—165 mg/dL
	Serum Creatinine—4.7 mg/dL

Serum CPKMB	11 U/L

Serum LDH	754.8 U/L

ANCA	**pANCA-Positive**
	Indirect immunofluorescence (IIF): perinuclear positivity
	ELISA (Euroimmun kit)-positive
	**cANCA-negative**

Anti-GBM	ELISA-negative
